# NGS-based barcoding with mini-*COI* gene target is useful for pet food market surveys aimed at mislabelling detection

**DOI:** 10.1038/s41598-020-74918-9

**Published:** 2020-10-20

**Authors:** Fabio Palumbo, Francesco Scariolo, Alessandro Vannozzi, Gianni Barcaccia

**Affiliations:** Laboratory of Genomics, Department of Agronomy Food Natural Resources Animals and Environment (DAFNAE), University of Padova, Campus of Agripolis, Viale dell’Università 16, 35020 Legnaro, PD Italy

**Keywords:** Biological techniques, Biotechnology, Genetics

## Abstract

Pet food industry has grown considerably in the last few years and it is expected to continue with this rate. Despite the economic impact of this sector and the consumer concerns for the increasing number of food and feed adulteration cases, few studies have been published on mislabelling in pet foods. We therefore investigated the capability of a next generation sequencing-based mini-barcoding approach to identify animal species in pet food products. In a preliminary analysis, a 127 bp fragment of the *COI* gene was tested on both individual specimens and ad hoc mixed fresh samples used as testers, to evaluate its discrimination power and primers effectiveness. Eighteen pet food products of different price categories and forms available on the market (i.e. kibbles, bites, pâté and strips) were analysed through an NGS approach in biological replicates. At least one of the species listed in the ingredients was not detected in half of the products, while seven products showed supplementary species in addition to those stated on the label. Due to the accuracy, sensitivity and specificity demonstrated, this method can be proposed as food genetic traceability system to evaluate both the feed and food quality timely along the supply chain.

## Introduction

Pet food is defined by the European Union as any product intended for the oral feeding of pet animals, including dog chews^1^. According to the European Pet Food Federation (FEDIAF), the trade body representing the European pet food industry, 80 million European households own at least one pet animal^2^. Therefore, it is not surprising that the European pet food industry is growing at an annual rate of 2.5% and that the annual sales volume in 2018 was as high as 8.8 million tons, with a turnover of 21 billion euros^2^. The same year, in the USA, pet food sales reached an all-time high of $31.68 billion^3^. More than 130 pet food companies are operating in the EU market^2^, and production is strictly regulated at every stage. The basic principles for feed and food safety are reported in Reg (CE) 178/2002^4^, which outlines the general principles of food safety, and in Reg (CE) 183/2005 on food and feed hygiene^5^. More-specific animal-by-product regulations^1,6^ were then adopted to regulate the use of raw materials of animal origin in pet food production and to set the health requirements necessary to import pet food products into the EU. Finally, considering that labelling represents the most important communication tool between food producers and pet owners, Reg (EC) 767/2009 “on the placing on the market and use of feed” was adopted to meet the urgent need to regulate this component of the industry^7^. One of the focuses of this latter regulation is to improve and modernize feed labelling in order to provide the necessary information to purchasers in a *consistent, coherent, transparent and understandable* way. Moreover, to better address this issue, the FEDIAF, with direct encouragement from the EU Commission, developed in 2018 a detailed ‘Code of good labelling practice for pet feed’^8^. According to the legal labelling requirements stated in the abovementioned Code, all feed materials should be listed indicating the name of each feed material in descending order by weight. Moreover, the percentage of weight should be indicated in the label, especially if the presence of a specific component is emphasised on the labelling in words, pictures or graphics^8^.

Food or feed labelling is therefore expected by the consumer to mirror the true identity of the product. However, despite existing regulations, food supply globalization and the exponential increase in international trade have amplified the potential for fraud (if deliberate and intentional) and accidental mislabelling to occur^9^. In fact, hundreds of studies have uncovered mislabelling practices in all the main food categories, including fish products^10^, meat and poultry derivatives^11^, spices^12^, coffee^13^ and cheese^14^.

A revolutionary step forward in promoting greater traceability and transparency was made with the advent of DNA barcoding, a molecular technique based on the DNA-level identification of differences that univocally characterize individual species^15^. Since the launch of the DNA barcoding initiative^16^, more than 500 peer-reviewed documents pertaining to DNA barcoding applied to food traceability have been published^17^. The spread of next-generation sequencing (NGS) platforms has further improved the potential of this technique, speeding up the possibility of simultaneously analysing multiple ingredients from complex matrixes^18^. Additionally, focusing the analysis on shorter hypervariable DNA sequences (e.g., 100–200 bp), designed within the full-length of barcoding genes, it is possible to acquire taxonomic information also from highly processed specimens characterized by fragmented DNA. This technique, known as DNA mini-barcoding, has been successfully used in several fields^19–22^.

Although DNA barcoding has been largely applied to different food sectors, studies on the traceability and mislabelling of pet foods are still scarce and generally performed on single species-based products using direct Sanger sequencing^23–26^ or multiple species with qPCR assays^9^. To the best of our knowledge, the study by Xing et al. represents the first (and, so far, only) attempt to investigate the species composition of pet food products by means of NGS-based DNA metabarcoding^27^, even though almost all of the products analysed (26 of 27) were for human consumption and the species within the products were limited to the Aves and Mammalia classes. Overall, these studies highlighted an impressive rate of pet food mislabelling that ranged from 38%^9^ to 100%^24^.

Therefore, to address the need for further research in this area, we tested the efficiency of the DNA barcoding approach combined with NGS in the reliable identification of a broad spectrum of species in pet food products. Moreover, to represent the most frequent store-bought pet food products, both dry and wet canned forms were considered. Finally, considering that frequent DNA degradation phenomena during food processing may limit the recovery of full-length sequence barcodes^25^, mini-barcode sequences were evaluated as a possible solution.

## Results

### DNA extraction and *COI* degenerate primer testing

Overall, the total gDNA extracted from the 10 fresh meat samples, 18 discount/premium pet food (PF) products and ad hoc mixtures (35 samples in total, including the replicates a and b, when available), had a mean concentration of 819 ng/µL and absorbance ratios at 260/280 nm between 1.5 and 1.8. As expected, DNA integrity was very poor in the pet food samples, with most products having DNA fragments in the gel of 300 bp or less, but consistently higher in the fresh meat samples and ad hoc mixtures, where the molecular lengths were over 10 kb.

Preliminary amplifications performed on fresh samples of 10 animal species demonstrated the effectiveness and specificity of the miniCOI primer pair in producing the target PCR products and, thus, single-band fragments on the agarose gel. PCR amplification failed only for shrimp (*Pandalus borealis*), the only species selected from the Malacostraca class. To further confirm the correspondence between the target sequence and the resulting amplified band, PCR products were Sanger sequenced: over an alignment length of 127 bp, 55 polymorphic sites (43% of the total) were detected (Supplementary Fig. 1). The number of SNPs discriminating samples from the same family (e.g., chicken and turkey, Phasianidae family) was 15 (of 127).

**Figure 1:**
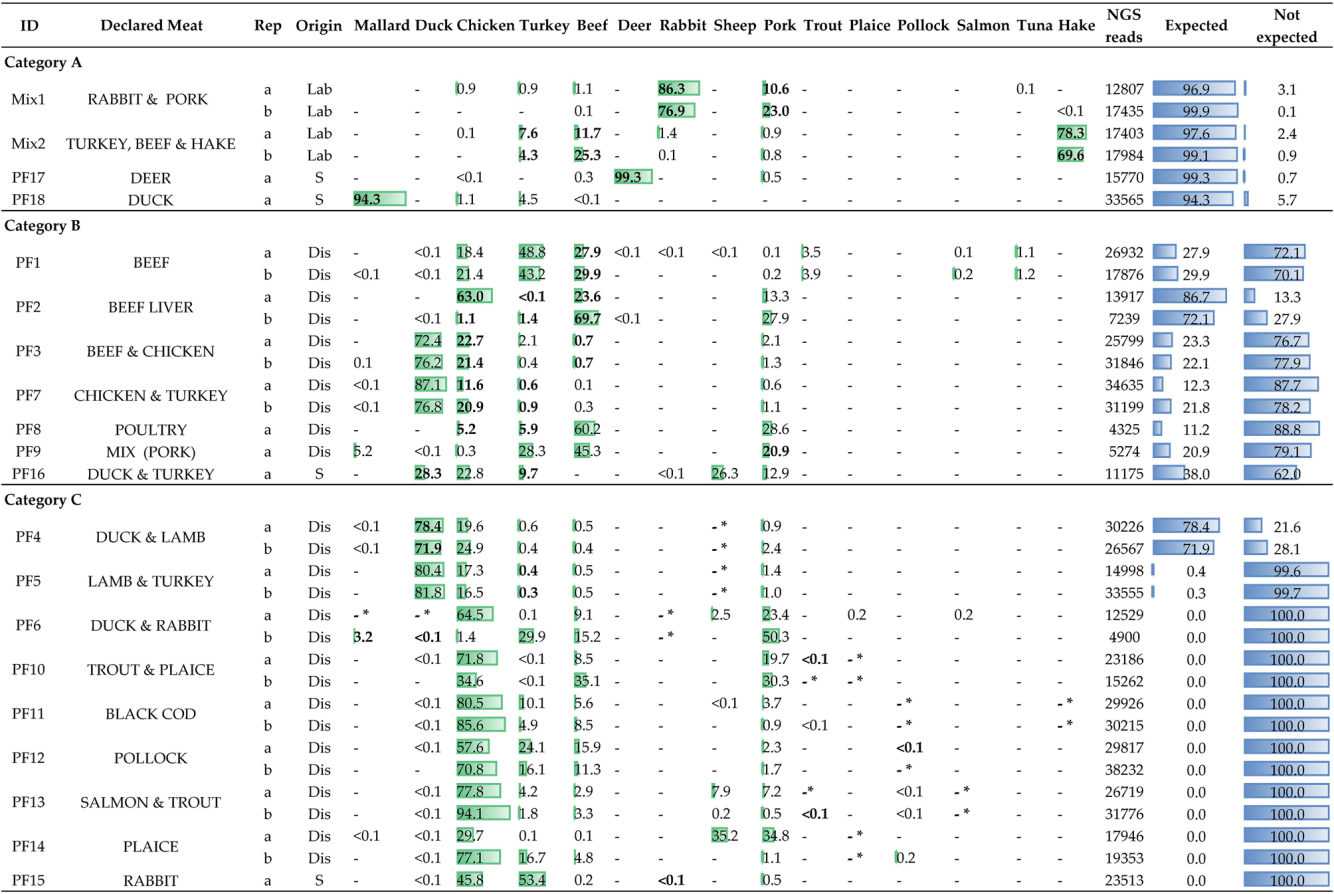
Summary of NGS results. The sample ID (ordered by category), meat types stated on the label, biological replicate and origin (Lab = assembled in the laboratory, Dis = discount store, S = supermarket) are shown in the first four columns. The relative abundances (%) of the 15 main species detected in the survey are indicated for each sample (and graphically represented by green bars), highlighting the expected species (according to the label) in bold when detected or with * when undetected. Finally, the number of NGS reads obtained for each sample and the overall abundance (%) of reads belonging to expected and not expected species are reported in the last two columns (and graphically represented by blue bars).

### NGS results for pet food products

The NGS approach was successfully applied to 35 samples (2 ad hoc mixtures, 18 commercial products and their biological replicates), and 734,110 paired-end sequences were retained. Except for one sample (PF8-b), which was excluded from the analyses due to a small number of reads (209), the samples exhibited an average number of reads per sample of 21,585, ranging from 4235 (PF8-a) to 38,232 (PF12-b). By means of a BLASTn search, reads were assigned to 15 species, whose relative abundances (%) are reported, for each sample, in Fig. 1.

From a qualitative point of view, the deep sequencing of the two ad hoc mixtures containing equal amounts of rabbit and pork meats (Mix1) or equal amounts of turkey, beef and hake tissues (Mix2) confirmed the presence of the expected species (Table 1). Additionally, the biological replicates of each ad hoc mixture showed comparable results. From a quantitative point of view, the relative abundances of each species deviated from the expected results. This was particularly evident for the ad hoc mixture containing rabbit (50%) and pork (50%) meats, where 81.6% (± 7%) of the reads were attributed to rabbit and only 16.8% (± 7%) to pork. Moreover, on average, 1.6% (± 1%) of the reads (considering the four replicates) were assigned to species other than those used for the preparation of the two mixtures (Fig. 1).

**Table 1:** Summary of samples analysed in this study. The sample ID, meat types advertised on the main label, meat-based ingredients listed on the label (ordered from most abundant to least abundant and, when available, also the percentage of weight), product type.

Sample ID	Publicized meat	Meat-based ingredient list	Product type	Intended use	Origin	Biological replicates
Mix1	–	Rabbit and pork (equal amounts)	ahM	–	Lab	2
Mix2	–	Turkey, beef and hake (equal amounts)	ahM	–	Lab	2
PF1	Beef	Beef 12%	K	D	Dis	2
PF2	Beef and liver	Beef 5%, liver 5%	P	C	Dis	2
PF3	Beef and chicken	Beef 5%, chicken 5%	B/S	C	Dis	2
PF4	Duck and lamb	Duck 5%, lamb 5%	B/S	C	Dis	2
PF5	Lamb and turkey	Lamb 5%, turkey 5%	B/S	C	Dis	2
PF6	Duck and rabbit	Duck 5%, rabbit 5%	P	C	Dis	2
PF7	Chicken and turkey	Chicken 5%, turkey 5%	B/S	C	Dis	2
PF8	Poultry	Chicken min. 5%, turkey min. 5%	P	C	Dis	2
PF9	Meat mix	Meat 42% (pork 8%), eggs and deriv	B	D	Dis	1
PF10	Trout and plaice	Meat and fish (trout 5%, plaice 5%)	P	C	Dis	2
PF11	Black cod	Meat and fish (black cod 5%)	B/S	C	Dis	2
PF12	Pollock	Meat and fish (pollock 5%)	B/S	C	Dis	2
PF13	Salmon and trout	Meat and fish (salmon 5%, trout 5%)	B/S	C	Dis	2
PF14	Plaice	Meat and fish (plaice 5%)	B/S	C	Dis	2
PF15	Rabbit	Rabbit	P/V	C	S	1
PF16	Duck and turkey	Duck and turkey	ST	C	S	1
PF17	Deer only	Deer only	P	D	S	1
PF18	Duck only	Duck only	P	C	S	1

ahM, ad hoc mixture; B, bites; B/S, bites with sauce; K, kibble; P, pâté; P/V, pâté with vegetables; ST, strips with vegetables in gelatin); intended use (D, dog food; C, cat food); origin (Lab, assembled in the laboratory; Dis, discount store; S, supermarket) and number of biological replicates analysed are reported.

NGS results derived from the 18 pet food products analysed were clustered into three categories: (A) samples whose content is represented, in percentages > 90%, by declared species, (B) samples displaying the declared species along with other species not expressly stated on the label and in percentages higher than 10%, and (C) samples lacking one or more of the declared species (Fig. 1).

The first category (A) included only 2 premium samples (i.e. both collected from supermarkets). Almost all of the reads produced for PF17 and PF18 were assigned to *Cervus elaphus* (99.3%) and *Anas platyrhynchos* (94.3%), respectively, in accordance with what was stated on the label (“only deer” and “only duck”, respectively).

Category B was represented by 7 products, 6 purchased from discount stores and 1 from a supermarket. In addition to the declared species, which were detected with relative abundances ranging from 0.3 to 78.4%, these products systematically contained other taxa not expressly stated on the label. Among these other taxa, *Gallus gallus*, *Meleagris gallopavo*, *Carina moschata, Bos taurus* and *Sus scrofa* were often recorded, with percentages ranging from 12.9 to 76.2%. For example, even though the abundance of beef sequences (28.9% ± 1%) found in both biological replicates of PF1 mirrored what was stated on the label (i.e., beef minimum of 12%), significant amounts of reads attributable to turkey (46.0% ± 4%) and chicken (19.9 ± 4%), both undeclared, were also observed. A similar pattern was observed for the only premium product included in the B category. In fact, PF16, in addition to the two labelled species of duck (28.3%) and turkey (9.7%), also contained consistent percentages of lamb (26.3%), chicken (22.8%) and pork (12.9%).

The last category (C) was represented by 9 products, including one premium pet food (PF15). In these samples, one or more species declared on the label were not detected. This category included, for instance, PF4: despite duck and lamb being advertised as the two main ingredients, the second species was not found in either of the biological replicates, which instead contained comparable amounts of duck (75.1% ± 5%) and chicken (22.2% ± 5%). Overall, the seven products in this study declaring rabbit (2) or any type of fish (5) were all grouped in this category, since the relative abundances of these species were always < 0.2%. Curiously, within samples, the number of reads assigned to *Gallus gallus* was always very high (29.7–94.1%), despite its presence never being mentioned on the ingredient list.

## Discussion

Traceability, now more than ever, represents an extremely popular topic in food supply chain research and, more generally, in the production industry. New techniques and methods for DNA barcoding have been developed in recent years and widely applied to different food sectors. However, although European pet food industry turnover is estimated to be approximately 21 billion euros/year^2^, with 80 million pet holders affected, only a few studies have focused on the univocal identification/authentication of animal species and mislabelling of animal food products. From the perspective of a possible routine application of this method, we tested the efficiency of NGS-based DNA barcoding approaches for the reliable detection of a broad spectrum of species in pet feeds. Short fragments (100–300 bp) of genomic DNA derived from products subjected to strong mechanical, physical and chemical processes are frequently degraded^28–30^, making it impossible to exploit the ~ 650 bp portion of cytochrome oxidase subunit 1 (*COI*), universally recognized as the gold standard for the detection of animal species^31–33^. To overcome this limitation, analyses were restricted to a 127 bp hypervariable region of the same gene using a degenerate version of a primer pair originally proposed by Meusnier et al.^34^. The results were encouraging since in the validation step, PCR amplicons were obtained for 9 of 10 samples belonging to three different classes, namely, Aves, Mammalia, and Actinopterygii (phylum Chordata). Amplification was unsuccessful only for *Pandalus borealis* (class Malacostraca). Although we did not investigate further the possible causes of this failure (because none of the pet food products subsequently analysed declared the presence of crustaceans), this finding is not surprising if we consider that, compared with the first three classes, this class belongs to a different phylum (Arthropoda). Moreover, it has been shown that crustaceans (to which the Malacostraca class belongs) show higher sequence variation in the *COI* mtDNA barcoding region than many other animal groups^35,36^. Albeit further studies are needed, a high polymorphism level in the primer annealing region could explain the failure of PCR amplification and, thus, the wide use of primers more specific to the Crustacea subphylum^37,38^. Sanger sequencing of the 9 PCR amplicons followed by multiple alignment confirmed the discriminatory power of the *COI* region used in this work.

One of the critical steps in the DNA barcoding pipeline is the extraction of adequate amounts of high-quality DNA from heterogeneous food matrixes^39^. In this work, different types of dry and wet matrixes were analysed (kibble, pâté, bites with sauce, and strips), all of which are the result of technological processes such as mechanical, thermal, chemical and enzymatic treatments. In such cases, specific commercial DNA extraction kits and customized DNA extraction protocols^30,40^ are required to ensure the isolation of high-quality DNA. In this study, the protocol described by Lagisz et al.^41^ was tested, resulting in good efficiency in terms of DNA quantity and the 260/280 ratio. The 260/230 ratio was instead lower (1.1 on average) than expected (2–2.2), probably due to the presence of large amounts of sugars and preservatives that characterize highly processed food products or beverages^13,42–44^. Due to the abovementioned production processes, DNA integrity was also compromised, with most products having DNA fragments in the gel of 300 bp or less, confirming the need for a mini-barcoding approach.

As a preliminary step to the analysis of commercial products in this study, two mixtures composed of fixed amounts of different animal tissues were successfully tested to define the NGS-based mini-barcoding efficiency and test the quantitative capability of the method. The results accurately mirrored the qualitative species composition, therefore verifying the strength potential of the primer pair proposed in this study. Only a small percentage of reads (e.g., < 1.4% rabbit in Mix2-a) was found to match unexpected species, which can be attributable to cross-contamination events that occurred either during mixture preparation (in the laboratory) or during meat processing (the butcher’s shop). However, owing to all sterility precautions adopted during mixtures preparation (i.e., use of laminar flow hood and sterile scalpels), the detection of unexpected species is likely due to contaminations during the slicing procedures (i.e. same knife used for different meat species) or contaminations by direct touching between parts of distinct species (i.e. direct contact between different meat pieces) in the local retailer where the products were purchased.

In contrast, pronounced quantitative bias was found since the species abundances extrapolated from the NGS data substantially deviated from the expected values. Even though the use of degenerate primers seems to considerably reduce this bias^45^, this finding is usually ascribed to the annealing efficiency of the primers and, in turn, differential amplification during NGS library preparation. Several authors documented technical challenges such as PCR bias and sequencing artefacts that strongly impacted the abundance of reads produced in metabarcoding studies^46–49^, and the overall solution is to cautiously examine all the sequencing-based quantitative estimates. Another possible explanation for this quantitative bias could be the quality of species-specific gDNA extracted from the mixtures, which could not be determined and may have impaired PCR amplification during library preparation.

Eighteen pet food products were chosen to represent (1) most of the dry and wet forms available on the market, (2) cat and dog foods, (3) products publicized as having single- or multispecies formulations, and (4) different price categories (i.e., discount and premium products). Overall, NGS data from biological replicates showed high reproducibility: among the 12 samples analysed in duplicates, 10 proved a qualitative consistency (i.e. both replicates displayed the same species) and 8 also matched from a quantitative point of view (i.e. both replicates displayed the same species with the same relative abundances).

Based on the correspondence between NGS results and labelling, the 18 pet food products analysed were clustered into three categories The only two products (category A) containing the species reported on the label in percentages higher than 90% both belonged to the “premium” category. This relates to the trust of consumers who are willing to spend up to three times more (prices reported in Materials and Methods) for a product whose content faithfully reflects what is reported on the label.

Overall, adulteration is defined as the act of either adding foreign substances or partly/wholly reducing essential nutrients^50,51^. The 7 pet food products showing (with percentages higher than 10%) supplementary species in addition to those stated on the label (formerly defined as category B) may be considered cases of unintentional adulteration due to negligence, contamination or accidental addition^27,52^. This is particularly true for the samples (e.g., PF2 and PF16) in which the species stated on the label were more abundant than those not included in the ingredient list. However, it seems unlikely that the species composition of some category B samples was simply the result of accidental adulteration. For instance, in both biological replicates of PF3 and PF7, more than 75% of the reads belonged to unlabelled species. Even if the quantitative estimates of an NGS approach must be handled with caution, situations like this deserve further investigation, especially when the economic value of undeclared species is lower than that of expected ones.

Due to the lack of one or more declared species, the last category (C) included the 9 pet food products for which adulteration and thus mislabelling were extensive. The preliminary analyses performed both on individual samples and on ad hoc mixtures to test the robustness of this method reasonably lead us to think that the lack of detection of some species was not dependent on the technique adopted. Moreover, it is worth highlighting that the vast majority of category C products advertised the presence of species (e.g., lamb or fish species) whose economic value is overall higher^53,54^ than that of other species more commonly used for food and feed preparation (e.g., chicken^55^ or pork^56^). As already widely reported for products intended for human consumption^57–59^, there is economic gain and greater market appeal in replacing expensive species with cheaper ones.

Pet owners should be able to be confident in both the safety and quality of commercial animal food products, but the growing number of recalls may erode the public's trust in the pet food industry and pet food products^15^. The most recent annual report (2018) released by the European Union related to this concept provides profound insight into the activity of the Rapid Alert System for Food and Feed (RASFF) and reveals that notifications regarding feed (and thus pet food) represent approximately 9% of the total notification volume, a percentage considerably larger than in previous years^60^. Albeit this growing concern, only few studies have been focused on the prevalence of mislabelling in pet foods. At this aim, DNA-based techniques for species detection may play a crucial role in providing concrete answers. Specifically, in this work, we demonstrated the high accuracy, sensitivity and specificity of an NGS-based mini-barcoding approach that can therefore serve as a food traceability system to evaluate both feed and food quality along the supply chain. Additional studies are certainly needed to further validate the primer couple here exploited for the first time, also expanding the number of meat/fish species to be tested. Future areas of work should also include the evaluation of the quantitative aspect of this technique, with particular attention to its detection limits.

## Materials and methods

### Primer choice

Twenty-six GenBank sequences of the ~ 650 bp mitochondrial cytochrome c oxidase I (*COI*) gene segment universally employed for DNA barcoding were retrieved from as many species belonging to the Mammalia (families: Bovidae, Cervidae, Leporidae and Suidae), Aves (families: Anatidae and Phasianidae), Actinopterygii (families: Gadidae, Merlucciidae, Pleuronectidae, Salmonidae, and Scombridae) and Malacostraca (family: Palaemonidae) classes. The sequences, which were chosen because they identified a variety of mammalian, avian, fish and crustacean species usually represented within the pet food market, were aligned using ClustalW in MEGA7^61^. Considering the difficulties in amplifying a 650 bp sequence from ultraprocessed pet food products (because of DNA degradation), the analyses were restricted to an ~ 170 bp (including primers) fragment of the gene. This was achieved by taking advantage of a primer pair originally proposed by Meusnier et al.^34^, with some modifications. In fact, according to the 26-sequence alignment, primers were improved by introducing degenerate bases to the 3′ end of both oligos. Thus, all the following analyses were performed using miniCOI-F: TCCACTAATCAYAARGAYATYGGHAC and miniCOI-R: GAAAATCATAATRAADGCRTGDGC.

### Primer testing on raw materials

Tissues of cow (*Bos taurus*), pork (*Sus scrofa*), rabbit (*Oryctolagus cuniculus*), chicken (*Gallus gallus*), turkey (*Meleagris gallopavo*), hake (*Merluccius gayi*), shrimp (*Pandalus borealis*), Atlantic salmon (*Salmo salar*), yellowfin tuna (*Thunnus albacares*) and European plaice (*Pleuronectes platessa*) were purchased from local retailers. Total genomic DNA, extracted according to the salting-out procedure described by Lagisz et al.^41^, was later quantified by spectrophotometric analysis (Thermo Fisher Scientific, Inc., Waltham, MA, United States). DNA integrity was checked by electrophoresis in a 1% agarose/1 × TAE gel containing 1 × SYBR Safe DNA Gel Stain (Life Technologies, Carlsbad, CA, USA). The efficiency of miniCOI primers was tested through PCR and Sanger sequencing. Amplification was carried out by means of a Veriti 96-Well Thermal Cycler (Applied Biosystems, Foster City, CA, USA), in a total volume of 25 μL of reaction mixture including 12.5 μL of MangoMix (Bioline, London, UK) with 1 μL of DNA (50 ng/μL), 2 μL of each primer (10 mM) and enough sterile water to reach the total volume. The following thermal conditions were adopted: 2 min at 95 °C, followed by 35 cycles at 95 °C for 30 s, 53 °C for 30 s, and 72 °C for 30 s and a final extension at 72 °C for 10 min. The PCR products were confirmed using 2% agarose/1 × TAE gels containing 1 × SYBR Safe DNA Gel Stain (Life Technologies), purified with ExoSAP-IT PCR Product Cleanup Reagent (Thermo Fisher) and sequenced on an ABI 3730XL Genetic Analyzer (Applied Biosystems).

All sequencing files were analysed using Geneious software v7.1.5 (Biomatters, Ltd., Auckland, New Zealand; https://www.geneious.com/) and queried using the Barcode of Life Database (BOLD) (https://www.boldsystems.org/) and GenBank (BLAST; https://blast.ncbi.nlm.nih.gov/Blast.cgi).

### NGS of ad hoc DNA mixtures and pet food products

First, to test the efficiency and effectiveness of the following NGS analyses, two ad hoc meat mixtures were produced under laminar flow hood, using sterile single use scalpels, each with two biological replicates. Both biological replicates of each mixture were prepared starting from the same original tissue samples. One mixture was composed of equal quantities (7.5 g) of rabbit and pork meats, while the second mixture was composed of equal amounts (5 g) of turkey, beef and hake tissues (Table 1).

Eighteen commercial pet food products (Table 1) obtained from Italian discount stores (14 products) and supermarkets (named “premium” products, 4 products) were purchased and analysed, when possible, with two biological replicates (from two different products). Products retrieved from discount stores were selected as the cheapest ones, with a cost lower than 3 €/kg, whereas the supermarket products, considered high-value products, had a selling price over 8 €/kg. The 18 products were also chosen to represent (1) most of the dry and wet forms in which pet foods are sold (i.e., kibble, bites, pâté, and strips), (2) products for cats and dogs, and (3) products publicized as singlespecies (n = 9) or multispecies (n = 9) pet food (Table 1).

15 g of each ad hoc mixture and pet food product were weighed into 50 mL Falcon tube and homogenized for 2 min with a T 10 basic Ultra Turrax homogenizer (IKA, Staufen, Germany). Total gDNA extraction was performed on 1.5 g of each homogenized sample, based on the protocol from Lagisz et al.^41^. The quality of each DNA extraction was checked as previously described for raw materials, through spectrophotometric analysis and agarose gel electrophoresis.

The miniCOI primer pair was used for the first step of amplicon library construction. A total of 2.5 µl of each DNA template (5–20 ng/µl) was amplified in a 25 µl PCR with 5 µl of 5X Flexi Buffer (Promega, Inc., Madison, WI, USA), 0.125 µl of GoTaq DNA Polymerase (5 U/μl, Promega, Inc.), 2 µl of primer mix (10 µM) and enough water to reach the total volume. PCR was executed in a GeneAmp PCR System 9700 (Thermo Fisher Scientific) with the following cycling conditions: a denaturation step at 95 °C for 2 min, followed by 40 cycles at 95 °C for 15 s, 52 °C for 15 s, and 72 °C for 30 s and a final extension step performed at 72 °C for 5 min. Strictly following the procedure described by Collier et al.^62^, the PCR products were checked on a 1.5% agarose gel, purified, equipped with dual indexes and Illumina sequencing adapters and finally sequenced on the Illumina MiSeq (PE300) platform (Illumina, Inc., San Diego, CA, USA).

### Bioinformatic analyses

Raw sequences were demultiplexed according to the Illumina indexes, assigned to samples and saved in FASTQ files. The MICCA pipeline^63^ was used for the quality check of the reads, their filtering and clustering into Operational Taxonomic Units (OTUs). Specifically, overlapping PE sequences were merged using the “mergepairs” command and reads statistics were calculated using the “stat” function. miniCOI primers were trimmed and reads were filtered using “preproc” (quality threshold = 20; length > 80 bp). Chimeric reads > 200 bp were removed too. The resulting sequences were then clustered into operational taxonomic units (OTUs) at 99% identity using the “otu” command. OTU assignment was performed by means of a manual BLASTn search based on the MegaBLAST algorithm and a local database was built using 356,149 *COI* sequences retrieved from GenBank. These sequences were selected because (1) also characterized by a BOLD ID (2) representing all the species available for Mammalia (3159 species), Aves (5233 species) and Actinopterygii (18,942 species). Species names were assigned if the best match score with a query OTU was ≥ 99%.

## Supplementary information

Supplementary Information.

## Data Availability

The data that support the findings of this study are available from the corresponding author upon request.
