# Supplementary material for: NGS-based barcoding with mini-COI gene target is useful for pet food market surveys aimed at mislabelling detection
Source: Sci Rep. 2020 Oct 20;10:17767. doi: 10.1038/s41598-020-74918-9 (PMC7575603; doi:10.1038/s41598-020-74918-9)
Supplement: Supplementary file 1 — Supplementary Information. [file 41598_2020_74918_MOESM1_ESM.pdf]

NGS-based barcoding with mini-*COI* gene target is useful for pet food market surveys aimed at mislabelling detection

Fabio Palumbo[1,§], Francesco Scariolo[1,§], Alessandro Vannozzi[1], Gianni Barcaccia[*,1]

Supplementary Files

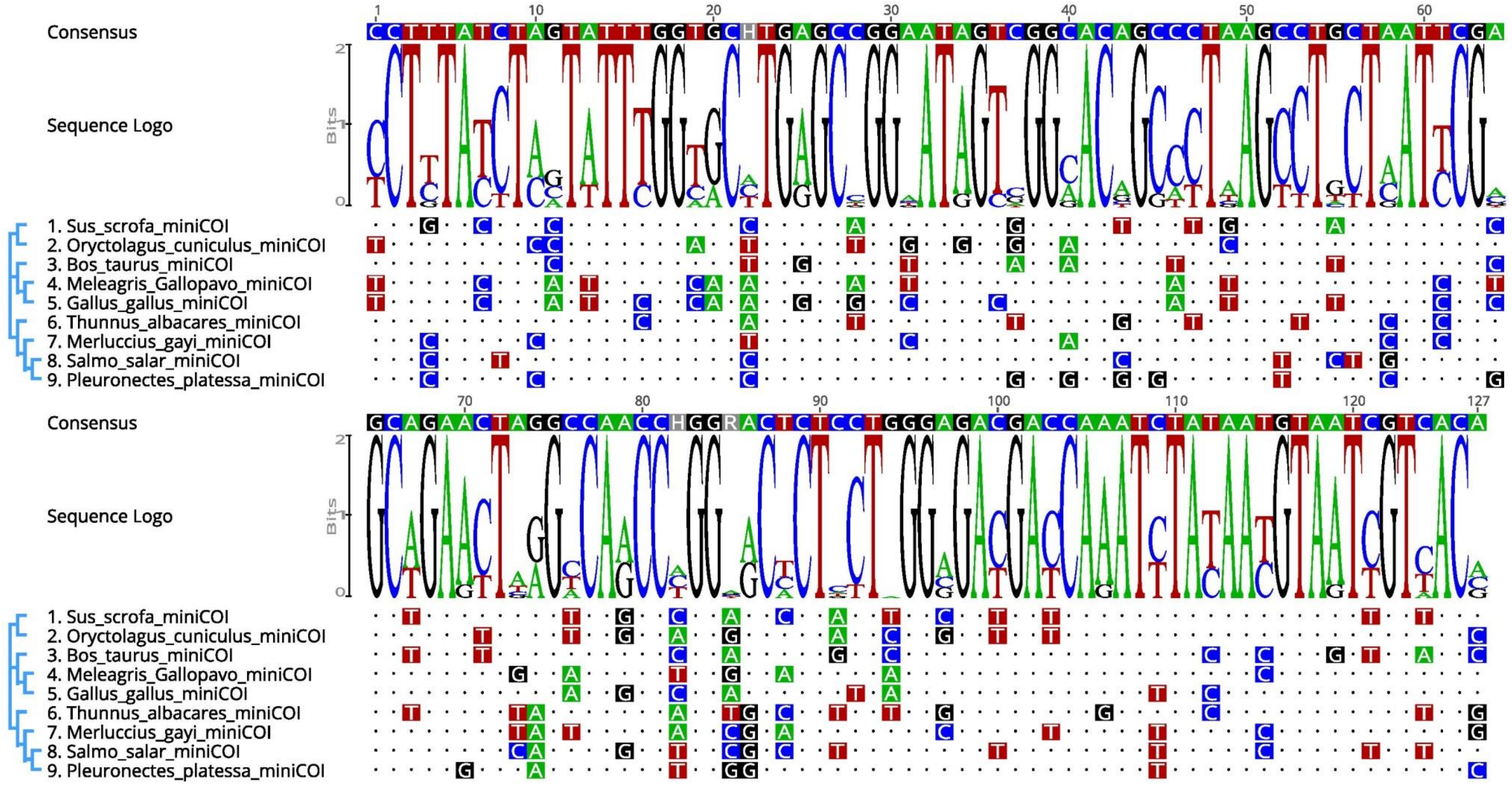

Supplementary Figure 1. Nucleotide alignment of nine 127 bp *COI* fragments (amplified by means of degenerate primers) from as many individual samples collected in the current study. The alignment and resulting sequence logo highlight 55 polymorphic positions (compared to the consensus sequence); a neighbor-joining dendrogram (on the left), based on the Tamura-Nei model, was also produced. All sequencing files were analysed using Geneious software v7.1.5 (Biomatters, Ltd., Auckland, New Zealand; https://www.geneious.com/)
